# Retraction: RAB22A overexpression promotes the tumor growth of melanoma

**DOI:** 10.18632/oncotarget.28726

**Published:** 2025-05-19

**Authors:** Feng Su, Yifei Chen, Shilin Zhu, Fangfang Li, Shuang Zhao, Lisa Wu, Xiang Chen, Juan Su

**Affiliations:** ^1^Department of Emergency, Xiangya Hospital, Central South University, Changsha, China; ^2^Department of Pharmacy, Xiangya Hospital, Central South University, Changsha, Hunan, China; ^3^Department of Neurology, The Second Affiliated Hospital of Hunan University of TCM, Changsha, Hunan, China; ^4^Department of Dermatology, Xiangya Hospital, Central South University, Changsha, Hunan, China; ^5^Hunan Key Laboratory of Skin Cancer and Psoriasis, Xiangya Hospital, Central South University, Changsha, Hunan, China; ^6^Institute of Medical Science Research, Xiangya Hospital, Central South University, Hunan, China

**This article has been retracted:** This decision follows an investigation by Oncotarget, which revealed duplications in several figures. Specifically, wound healing assay images in Figures 4D and 5C are identical to images found in Figure 3B of Zhou et al. [1], which was published concurrently. The transwell assay image in Figure 4E duplicates images present in figure 4D of a retracted paper [2]. In addition, transwell assay images in Figures 4E and 5D were found in Figures 3C and 4C of reference [3]. Furthermore, blot images in panel 5A are identical to those in Figure 4A of another unrelated, concurrently published paper [4], which has also since been retracted. Consequently, the Editorial decision was made to retract this publication. The corresponding authors have indicated their agreement with this retraction. However, the editorial office has not received the formal retraction documentation signed by all authors.

Original article: Oncotarget. 2016; 7:71744–71753. 71744-71753. https://doi.org/10.18632/oncotarget.12329

## References

[1] Zhou W , Zou B , Liu L , Cui K , Gao J , Yuan S , Cong N . MicroRNA-98 acts as a tumor suppressor in hepatocellular carcinoma via targeting SALL4. Oncotarget. 2016; 7:74059–73. 10.18632/oncotarget.12190. 27677076 PMC5342035

[2] Liu W , Xiao P , Wu H , Wang L , Kong D , Yu F . MicroRNA-98 Plays a Suppressive Role in Non-Small Cell Lung Cancer Through Inhibition of SALL4 Protein Expression. Oncol Res. 2017; 25:975–88. 10.3727/096504016X14791726591124. . Retraction in: Oncol Res. 2021; 28:829. DOI: 10.3727/096504021X16207253542097 PMID: 34503608 27938506 PMC7841028

[3] Hu Z , Cui Y , Zhou Y , Zhou K , Qiao X , Li C , Wang S . MicroRNA-29a plays a suppressive role in non-small cell lung cancer cells via targeting LASP1. Onco Targets Ther. 2016; 9:6999–7009. 10.2147/OTT.S116509. 27895492 PMC5117897

[4] Xiao L , Zhou H , Li XP , Chen J , Fang C , Mao CX , Cui JJ , Zhang W , Zhou HH , Yin JY , Liu ZQ . MicroRNA-138 acts as a tumor suppressor in non small cell lung cancer via targeting YAP1. Oncotarget. 2016; 7:40038–46. 10.18632/oncotarget.9480. . Retraction in: Oncotarget. 2024; 15:590. DOI: 10.18632/oncotarget.28645 PMID: 39189975 27223073 PMC5129990

